# The Chicago Gynæcological Society

**Published:** 1879-01

**Authors:** 


### ARTICLE XIV.

### THE CHICAGO GYNÆCOLOGICAL SOCIETY.

On the evening of the 18th of October, 1878, a few gynæcologists of this city, by invitation, met at the house of Dr. W. H. Byford, to form a new medical society. Drs. DeLaskie Miller, A. Reeves Jackson, E. O. F. Roler, James H. Etheridge, T. Davis Fitch and H. W. Jones were present. Dr. Miller was called to the chair, and after an informal and somewhat lengthy discussion of the objects of the meeting, a resolution was passed to organize a society for the cultivation of knowledge in connection with obstetrics and diseases of women and children. In selecting a name suitable to represent the objects of the Society, the choice between an "Obstetrical" Society and a "Gynæcological" Society was freely canvassed. It was conceded that neither of these names was sufficiently comprehensive to embrace all the objects of the organization, but it was finally decided that the latter was capable of a wider application than the former; hence it was adopted, and the name, Chicago Gynæcological Society agreed upon.

The organization of the Society was completed by electing Dr. H. W. Jones secretary for one year. The secretary performs all the usual duties of that office, and, so far as necessary, exercises the functions of treasurer. There are no other permanent offices. The chairman is nominated and elected at each meeting. As yet there is no written constitution nor rules for the government of the Society, these being regarded as at present unnecessary. The meetings are held at the homes of the members by invitation, the last Friday of each month, at 8 p. m. precisely. The exercises of the evening consist of reading and discussing an appropriate paper once every three months, and at other meetings conversations upon such subject as may be agreed upon the evening previous. One member is selected at the time the topic for conversation is chosen, to lead upon the subject selected. After the discussion is closed, the members partake of a simple collation prepared by the host of the evening.

The first regular meeting was appointed to take place at the residence of Dr. H. W. Jones, on the evening of Friday the 25th of October. The following named gentlemen were invited to meet the Society at that time and place as original members, viz.: Drs. E. Warren Sawyer, Danl. T. Nelson, Henry P. Merriman, Chas. W. Earle, Henry T. Byford and E. C. Dudley.

At the second meeting it was resolved that candidates for membership should furnish the secretary a paper on some topic embraced in the declared object of the organization, and the recommendations of two members of the Society. At the meeting succeeding the nomination, the application must be accepted by a unanimous vote of all the members present.

We heartily wish success and prosperity to this new enterprise.

The fruits of the regular meetings of the Chicago Gynæcological Society are already seen in the reports published in the JOURNAL AND EXAMINER.

---

### EASTERN CORRESPONDENCE.

We take pleasure in announcing that we have completed arrangements by which we expect to be enabled to present to our readers, at regular intervals throughout the current year, correspondence from able writers in Boston, New York, and Philadelphia, which will embody such practical information and news of current events, as will add to the value of these pages.

---

### TELEPHONIC COMMUNICATION.

By the aid of the Edison-Gray telephone, and the ample facilities offered by the American District Telegraph Co., the members of the editorial corps of this journal will soon be within vocal distance of each other and their publishers, and will also be enabled to communicate, by the same instrumentality, with several of the hospitals and dispensaries of the city, and with an increasingly large number of professional men.

